# Structure and Chemical Bonding of the Li-Doped Polar Intermetallic *RE*_2_In_1−*x*_Li*_x_*Ge_2_ (*RE* = La, Nd, Sm, Gd; *x* = 0.13, 0.28, 0.43, 0.53) System

**DOI:** 10.3390/ma11040495

**Published:** 2018-03-26

**Authors:** Junsu Lee, Jieun Jeon, Tae-Soo You

**Affiliations:** Department of Chemistry, Chungbuk National University, Cheongju, Chungbuk 28644, Korea; ievei4567@naver.com (J.L.); fannyzza88@hanmail.net (J.J.)

**Keywords:** polar intermetallics, single crystal X-ray diffraction, site-preference of Li, electronic structure

## Abstract

Four polar intermetallic compounds belonging to the *RE*_2_In_1−*x*_Li*_x_*Ge_2_ (*RE* = La, Nd, Sm, Gd; *x* = 0.13(1), 0.28(1), 0.43(1), 0.53(1)) system have been synthesized by the traditional solid-state reaction method, and their crystal structures have been characterized by single-crystal X-ray diffraction (SXRD) analyses. The isotypic crystal structures of four title compounds adopt the Mo_2_FeB_2_-type structure having the tetragonal space group *P4/mbm* (*Z* = 2, Pearson code *tP*40) with three crystallographically independent atomic sites and can be simply described as a pile of the identical 2-dimensioanl (2D) *RE*_2_In_1-*x*_Li*_x_*Ge_2_ slabs stacked along the *c*-axis direction. The substituting Li atom shows a particular site preference for replacing In at the *Wyckoff* 2*a* site rather than Ge at the *Wyckoff* 4*g* in this crystal structure. As the size of a used rare-earth metal decreases from La^3+^ to Gd^3+^ throughout the title system, the Ge-Ge and Ge-In/Li bond distances, both of which consist of the 2D anionic Ge_2_(In/Li) layer, gradually decrease resulting in the reduction of a unit cell volume. A series of theoretical investigations has been performed using a hypothetical structure model Gd_2_In_0.5_Li_0.5_Ge_2_ by tight-binding linear muffin-tin orbital (TB-LMTO) method. The resultant densities of states (DOS) value at the Fermi level (*E*_F_) suggests a metallic conductivity for this particular composition, and this calculation result is in a good agreement with the formal charge distribution assigning two extra valence electrons for a metal-metal bond in the conduction band. The thorough analyses of six crystal orbital Hamilton population (COHP) curves representing various interatomic interactions and an electron localization function (ELF) diagram indicating the locations of paired-electron densities are also provided in this article.

## 1. Introduction

A family of intermetallic compounds belonging to the *RE*_5_*Tt*_4_ (*RE* = rare-earth metals, *Tt* = tetrels) series [1,2,3] have drawn worldwide attention during the last decade due to their extraordinary chemical and physical characteristics, such as giant magnetoresistance [4,5] and colossal magnetorestriction [6,7,8,9]. Among its members, a ternary compound Gd_5_Si_2_Ge_2_ [10] proves that the giant magnetocaloric effect (MCE) can possibly occur at near room temperature through the first-order magnetic phase transition between the ferromagnetic and paramagnetic phases as a function of temperature. Given that this interesting magnetic phase transition has been proven to be triggered by the crystal structure transformation between the orthorhombic and tetragonal crystal systems, numerous research activities have focused on understanding the correlation among composition-structure-property of the particular ternary solid-solution Gd_5_Si_2−*x*_Ge_2+*x*_ system [10,11], as well as various other *RE*_5_*Tt*_4_ series [12,13,14]. My research group has recently published an article about the isotypic *RE*_4_LiGe_4_ (*RE* = La, Ce, Pr, Sm) system [15], where a monovalent Li specifically substituted *RE* at the particular site among three available candidate sites. As a result of our thorough study, we claimed that a significant size difference between *RE* and Li generated a sufficient enough chemical pressure causing the observed structural transformation, and this transformation eventually resulted in modifying magnetic characteristics of these compounds.

During our systematic investigations, we serendipitously obtained four quaternary polar intermetallics belonging to the *RE*_2_In_1−*x*_Li*_x_*Ge_2_ (*RE* = La, Nd, Sm, Gd; *x* = 0.13(1), 0.28(1), 0.43(1), 0.53(1)) system, and all four title compounds crystallized in the Mo_2_FeB_2_-type structure [16]. Interestingly, this type of crystal structure is isotypic to the structural moiety of the 2-dimensioanl (2D) *RE*_2_LiGe_2_ slabs consisting of the parental *RE*_4_LiGe_4_ (*RE* = La, Ce, Pr, Sm) system [15]. As already mentioned in our previous article, the imaginary *RE*_2_LiGe_2_ compound adopting the Mo_2_FeB_2_-type structure could be derived from the parental *RE*_4_LiGe_4_ system by simply removing the interlayer Ge_2_ dimers bridging two neighboring *RE*_2_LiGe_2_ slabs [15]. In fact, large numbers of the Mo_2_FeB_2_-type analogues belonging to the *RE*_2_*MTt*_2_ (*RE* = rare-earth metals; *M* = Li, Mg, Al, Sc, Y, In; *Tt* = Si, Ge) family have already been reported due to their intriguing magnetic and electrical properties, and their crystal structures [17,18,19,20,21,22,23,24,25,26,27]. For instance, the magnetic property of Gd_2_MgGe_2_ [17] is successfully shifted by the non-magnetic Mg substitution for the magnetic Ni in Gd_2_NiGe_2_. In addition, the crystal structure of Rh_2_LiSi_2_ [28] contains the 3D anionic open-framework structure rather than the typical 2D anionic Mo_2_FeB_2_-type layered structure due to the size ratio between cationic and anionic elements. Although there are numerous reports about the ternary *RE*_2_*MTt*_2_ family, there is little report about quaternary derivatives belonging to this family. Therefore, we decided to conduct in-depth investigations into the title quaternary system in terms of experimental and theoretical perspectives.

In this article, we report the comprehensive research results of our systematic investigation about the monovalent Li substituted *RE*_2_In_2−*x*_Li*_x_*Ge_2_ (*RE* = La, Nd, Sm, Gd; *x* = 0.13(1), 0.28(1), 0.43(1), 0.53(1)) system. Our experimental studies include the high-temperature synthesis, crystal structure characterization by single-crystal X-ray diffraction (SXRD) analyses, and thorough structural discussion. Theoretical investigations performed by tight-binding linear muffin-tin orbital (TB-LMTO) using a hypothetical Gd_2_In_0.5_Li_0.5_Ge_2_ model provide the basis for detailed discussions about an overall electronic structure including densities of states (DOS), crystal orbital Hamilton populations (COHP) curves and an electron localization function (ELF) contour map.

## 2. Materials and Methods

### 2.1. Synthesis

All of the sample preparation processes were performed inside a N_2_-filled glovebox with O_2_ and H_2_O contents below 0.1 ppm or inside an arc-melting furnace under Ar atmosphere. The reactant elements were purchased from Alfa Aesar or Aldrich: La (pieces, 99.9%), Nd (ingot, 99.9%), Sm (pieces, 99.9%), Gd (chip, 99.9%), In (shot, 99.99%), Li (wire, 99.8%) and Ge (ingot, 99.9999%). Rare-earth metals and Li were cleaned by scraping off the lightly tanned surfaces using a scalpel or a metal brush inside a glovebox before loading in a Nb ampoule. Originally, the four title compounds in the *RE*_2_In_1−*x*_Li*_x_*Ge_2_ (*RE* = La, Nd, Sm, Gd; *x* = 0.13(1), 0.28(1), 0.43(1), 0.53(1)) system were serendipitously produced as side products during our investigations of the *RE*_4_LiGe_4_ (*RE* = La, Ce, Pr, Sm) system [15]. Once the crystal structures of the four products were determined as the Mo_2_FeB_2_-type structure based on the SXRD analysis, we attempted to reproduce these compounds by loading the refined stoichiometric compositions. In addition, we also tried to obtain the phase-pure products by changing the loaded compositions. However, all these reactions also produced some other secondary phases, such as Nd_3_Ge_5_, besides the title *RE*_2_In_1−*x*_Li*_x_*Ge_2_ phase.

Each reactant was cut into small pieces and loaded in one end-sealed Nb ampoule inside a glovebox, and the other end of the ampoule was sealed by arc welding under an Ar atmosphere. Then, the Nb ampoule was sealed again in a secondary container of a fused-silica jacket under vacuum to prevent a Nb ampoule from the oxidation during the high-temperature reaction process. The reactant mixtures were heated to 1353 K at a rate of 473 K/h, kept there for 5 h, and then cooled to 1023 K at a rate of 10 K/h, after which these products were annealed for two days. After that, the reactions were naturally cooled down to room temperature by turning off the furnace [15].

### 2.2. X-ray Diffraction Experiments

Four title compounds in the *RE*_2_In_1−_*_x_*Li*_x_*Ge_2_ (*RE* = La, Nd, Sm, Gd; *x* = 0.13(1), 0.28(1), 0.43(1), 0.53(1)) system were characterized by SXRD analyses. Data were collected at room temperature using a Bruker SMART APEX2 CCD-based diffractometer equipped with Mo Kα_1_ radiation (*λ* = 0.71073 Å). Initially, several silvery lustrous needle-shaped single crystals were chosen from each batch of products. After the quality check, the best single crystal was carefully selected, and a full data collection was conducted using Bruker’s *APEX2* program [29]. Data reduction, integration, and unit cell parameter refinements were executed using the *SAINT* program [30]. *SADABS* [31] was used to perform semiempirical absorption corrections based on equivalents. The entire sets of reflections of the four title compounds were in good agreements with the tetragonal crystal system, and the space group *P*4/*mbm* (No. 127) was chosen for the four isotypic polar intermetallics. The detailed crystal structures were solved by direct methods and refined to convergence by full matrix least-squares methods on *F*^2^. The refined parameters of these Mo_2_FeB_2_-type phases include the scale factor, atomic positions including anisotropic displacement parameters (ADPs), extinction coefficients, and occupancy factors of the In/Li-mixed sites. During the last stage of this structure refinement cycle, each atomic position was standardized using *STRUCTURE TIDY* [32]. Important crystallographic data, atomic positions with ADPs and several selected interatomic distances are provided in Table 1, Table 2 and Table 3. Further details regarding each crystal structure can be obtained from the Fachinformationszentrum Karlsruhe, 76344 Eggenstein-Leopoldshafen, Germany (fax: (49) 7247-808-666; e-mail: crysdata@fiz-karlsruhe.de)—under depository numbers CSD-433825 for La_2_In_0.72(1)_Li_0.28_Ge_2_, CSD-433826 for Nd_2_In_0.87(1)_Li_0.13_Ge_2_, CSD-433828 for Sm_2_In_0.57(1)_Li_0.43_Ge_2_ and CSD-433828 for Gd_2_In_0.47(1)_Li_0.53_Ge_2_.

### 2.3. Electronic Structure Calculations

To understand the electronic structures of the four title compounds, including individual chemical bonding and locations of paired electrons, a series of theoretical calculations were performed using a hypothetical structure model Gd_2_In_0.5_Li_0.5_Ge_2_ by the Stuttgart *TB-LMTO47* program [33,34,35,36,37] with atomic sphere approximation (ASA). Since the experimentally refined composition of the Gd compound is the closest to a practically accessible composition for calculations, we decided to exploit the Gd compound as a calculation model. To apply an idealized composition of Gd_2_In_0.5_Li_0.5_Ge_2_, we designed a $\sqrt{2}$ × 2 × $\sqrt{2}$ superstructure involving an alternating atomic arrangement of In and Li along the *ab*-plane as well as the *c*-axis directions in the expanded unit cell. For this superstructure model, the orthorhombic space group *Pmma* (No. 51) was exploited, rather than the crystallographically refined tetragonal space group *P*4/*mbm* (No. 127). Lattice parameters and atomic coordinates were estimated from the SXRD data of Gd_2_In_0.47(1)_Li_0.53_Ge_2_. Further details about this structure model are provided in Supporting Information (Table S1). In the ASA method, space is filled with overlapping Wigner-Seitz (WS) atomic spheres [33,34,35,36,37]. All relativistic effects, except spin-orbit coupling, were taken into account using a scalar relativistic approximation. The symmetry of the potential inside each WS sphere was considered spherical, and a combined correction was used to take into account the overlapping part. The radii of the WS spheres were obtained by requiring the overlapping potential be the best possible approximation of the full potential and were determined by an automatic procedure [33,34,35,36,37]. This overlap should not be too large, because the error in kinetic energy introduced by the combined correction is proportional to the fourth power of the relative sphere overlap. The used WS radii are listed as follows: Gd = 1.831 Å, In = 1.771 Å, Li = 1.711 Å and Ge = 1.343 Å for Gd_2_In_0.5_Li_0.5_Ge_2_. The basis sets included 6*s*, 6*p*, 5*d*, and *4f* orbitals for Gd; 2*s*, 2*p*, and 3*d* orbitals for Li; 5*s*, 5*p*, 5d and 4*f* orbitals for In; and 4*s*, 4*p* and 4*d* orbitals for Ge. The Gd 6*p*, In 5*d* and 4*f*, Li 2*p* and 3*d*, and Ge 4*d* orbitals were treated by the Löwdin downfolding technique [38]. The *k*-space integration was conducted by the tetrahedron method [39], and the self-consistent charge density was obtained using 343 irreducible *k*-points in the Brillouin zone.

## 3. Results and Discussion

### 3.1. Crystal Structure Analysis

Four polar intermetallic compounds belonging to the *RE*_2_In_1−*x*_Li*_x_*Ge_2_ (*RE* = La, Nd, Sm, Gd; *x* = 0.13(1), 0.28(1), 0.43(1), 0.53(1)) system were synthesized by the high-temperature reaction method, and their crystal structures were characterized by SXRD analyses. All four title compounds crystallized in the tetragonal space group *P*4/*mbm* (Pearson code *tP*20, *Z* = 2) and adopted the Mo_2_FeB_2_-type structure [16] (Table 1), which is a ternary version of the U_3_Si_2_-type structure [40]. There exist three crystallographically independent asymmetric atomic sites, each including one rare-earth metal site, one Ge site and one In/Li-mixed site (Table 2).

**Figure 1:**
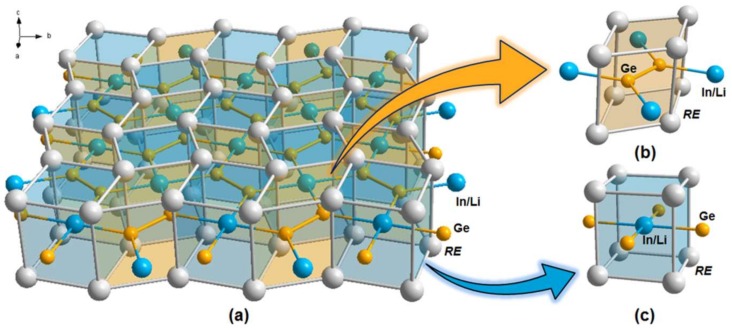
(**a**) The 2D infinite layered structure consisting of the overall crystal structure of the *RE*_2_In_1−*x*_Li*_x_*Ge_2_ (*RE* = La, Nd, Sm, Gd; *x* = 0.13(1), 0.28(1), 0.43(1), 0.53(1)) system. The crystal structure is represented by the combination of ball-and-stick and polyhedra representations, and viewed down the *c*-axis direction. The Ge_2_ dimers are highlighted in orange color. The local coordination geometry of (**b**) the *RE*Ge_2_ moiety (AlB_2_-type) and (**c**) the *RE*(In/Li) moiety (CsCl-type) are also illustrated. Color codes: *RE*, gray; Ge, orange; In/Li mixed-site, blue.

The overall isotypic crystal structures of the four title compounds can be simply described as a pile of identical 2D *RE*_2_In_1−*x*_Li*_x_*Ge_2_ slabs propagating along the *ab*-plane direction (Figure 1a). Each of these 2D slabs can be further considered as the 1:1 intergrowth of the *RE*Ge_2_ and *RE*(In/Li) moieties adopting the AlB_2_-type and CsCl-type structures, respectively. A Ge atom occupies the central site of a trigonal-prismatic polyhedron formed by six *RE* atoms (Figure 1b), whereas the In/Li-mixed site occupies the central site of a cubic polyhedron shaped by eight *RE* atoms (Figure 1c). Interestingly, the substituting Li atom shows a particular site preference for the In site (*Wyckoff* 2*a*), rather than the Ge site (*Wyckoff* 4*g*), as it produces this quaternary system. This type of site preference of Li resembles that of Li in the previously reported *RE*_4_LiGe_4_ (*RE* = La, Ce, Pr, and Sm) system [15].

**Figure 2:**
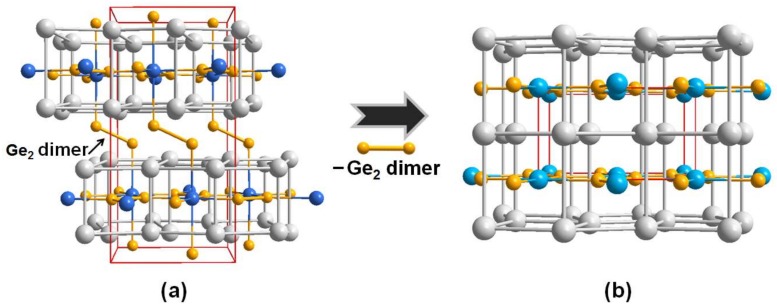
Schematic illustration showing the structural correlation between (**a**) the *RE*_4_LiGe_4_ (*RE* = La, Ce, Pr, Sm) system and (**b**) the *RE*_2_In_1−*x*_Li*_x_*Ge (*RE* = La, Nd, Sm, Gd; *x* = 0.13(1), 0.28(1), 0.43(1), 0.53(1)) system. A unit cell of each structure type is outlined in red, and the both of the interlayer and intralayer Ge_2_ dimers are highlighted in orange-color. Color codes: *RE*, gray; Ge, orange; In/Li mixed-site, blue.

The overall crystal structure of the *RE*_4_LiGe_4_ system can be considered as an assembly of two basic substructures: (1) the Mo_2_FeB_2_-type 2D infinite *RE*_2_LiGe_2_ slabs, and (2) the dumbbell-shaped Ge_2_ dimers bridging two of those neighboring 2D *RE*_2_LiGe_2_ slabs. Interestingly, the local structural geometry of the 2D *RE*_2_LiGe_2_ slab in the *RE*_4_LiGe_4_ system is nearly identical to that of the 2D *RE*_2_In_1−*x*_Li*_x_*Ge_2_ slab in our title compounds, which can be regarded as a building block establishing the overall crystal structure. One noticeable difference between these two structure types is the additional bridging Ge_2_ dimers in-between two neighboring 2D *RE*_2_LiGe_2_ slabs in the *RE*_4_LiGe_4_ system as illustrated in Figure 2.

**Figure 3:**
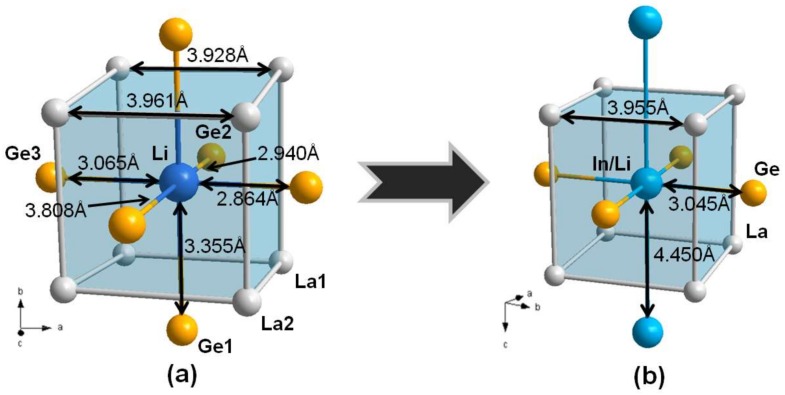
Local coordination geometries of the CsCl-type cubic-shaped (**a**) LaLi moiety in La_4_LiGe_4_ and (**b**) La(In/Li) moiety in La_2_In_0.72(1)_Li_0.28_Ge_2_. Selected interatomic distances are also displayed. Color codes: *RE*, gray; Ge, orange; In/Li mixed-site, blue.

Two Ge-Ge and Ge-In/Li bond distances in the 2D Ge_2_(In/Li) anionic layer and the local coordination geometries of structural moieties in the title *RE*_2_In_1−*x*_Li*_x_*Ge_2_ system can be compared with those observed in the known isotypic or homeotypic compounds, such as the *RE*_2_InGe_2_ (*RE* = La − Ho, Yb) [18,19] and *RE*_4_LiGe_4_ (*RE* = La, Ce, Pr, Sm) systems [15]. First, the Ge-Ge bond distance of the Ge_2_ dimer along the *ab*-plane direction in our title compounds ranges from 2.4998(12) to 2.5384(8) Å (Table 3), and this range of bond distances is very similar to that found in the isotypic *RE*_2_InGe_2_ (*RE* = La − Ho, Yb) system, ranging between 2.504 and 2.526 Å. This implies that the Li substitution for In nearby Ge hardly influences the given coordination environment of Ge in this system. In particular, these Ge-Ge distances in two of our title compounds La_2_Li_0.28(1)_In_0.72_Ge_2_ (2.5384(8) Å) and Sm_2_Li_0.43(1)_In_0.57_Ge_2_ (2.5252(14) Å) are also comparable to those in La_4_LiGe_4_ (2.549(1) Å) and Sm_3.98_Li_1.02_Ge_4_ (2.532(1) Å) [15]. Second, the Ge-In/Li distances along the *ab*-plane in four compounds varying from 3.0448(3) to 2.8985(9) Å are also comparable to the Ge-In distances ranging from 2.855 to 3.009 Å in the *RE*_2_InGe_2_ (*RE* = La − Ho, Yb) system [18,19]. However, four Ge-Li bond distances along the *ac*-plane direction in each of La_4_LiGe_4_ and Sm_3.98_Li_1.02_Ge_4_, which are the counterparts of the Ge-In/Li distance in this work, display substantial differences from one another between 3.808 and 2.864 Å and between 3.658 and 2.742 Å, respectively [15]. This type of rather large deviation of the Ge-Li distance could be attributed to the significantly distorted cubic *RE*Li polyhedra (CsCl-type) in La_4_LiGe_4_ and Sm_3.98_Li_1.02_Ge_4_, and therefore the four Ge-Li bonds connecting this *RE*Li moiety to the four neighboring *RE*Ge_2_ moieties can hardly be the same. On the other hand, the cubic *RE*(In/Li) polyhedra in the title compounds are relatively more symmetric and contain only one type of Ge-In/Li distance in each compound, as compared in Figure 3.

**Figure 4:**
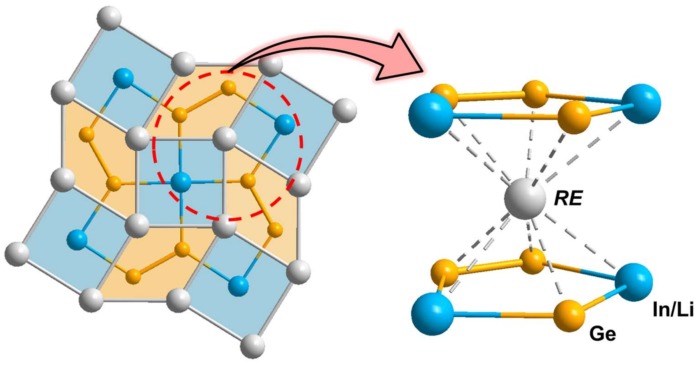
Distorted pentagonal-prismatic coordination environment surrounding a cationic *RE* site in the *RE*_2_In_1−*x*_Li*_x_*Ge_2_ (*RE* = La, Nd, Sm, Gd; *x* = 0.13(1), 0.28(1), 0.43(1), 0.53(1)) system.

Finally, the bond distances of Ge-Ge and Ge-In/Li consisting of the 2D anionic Ge_2_(In/Li) layer gradually decrease from 2.5384(8) to 2.500(1) Å and from 3.044(3) to 2.8985(9) Å, respectively, as the size of a cation decreases from La^3+^ to Gd^3+^ (*r*(La^3+^) = 1.27 Å for 10 coordinates; *r*(Gd^3+^) = 1.11 Å for 9 coordinates) [41] throughout the title system (Table 3). In addition, these bond shortenings influenced by the cationic-size reduction eventually result in the contraction of a unit cell volume (Table 1). This kind of intimate correlation among the bond distance, the cationic size, and the unit cell volume should be attributed to the slightly distorted pentagonal-prismatic coordination environment surrounding a cationic *RE*, which is formed by a total of ten anionic elements, including six Ge and four In/Li-mixed sites (see Figure 4). Table 3 also shows the declining trend of these *RE*-*RE* and *RE*-In/Li distances along the four title compounds.

### 3.2. Electronic Structure and Chemical Bonding

To understand the overall electronic structure of the *RE*_2_In_1__−_*_x_*Li*_x_*Ge_2_ (*RE* = La, Nd, Sm, Gd; *x* = 0.13(1), 0.28(1), 0.43(1), 0.53(1)) system, including interatomic interactions and locations of paired electron densities, we conducted a series of theoretical calculations by the TB-LMTO-ASA method [33,34,35,36,37] and analyzed DOS and COHP curves, as well as an ELF contour map. For practical reasons, we should use a structural model that employs an idealized composition of Gd_2_In_0.5_Li_0.5_Ge_2_ with an alternating In and Li arrangement along the *ab*-plane and the *c*-axis directions in a unit cell. Therefore, the √2 × 2 × √2 superstructure with a space group of *Pmma* was designed, and lattice parameters and atomic positions extracted from SXRD data of Gd_2_Li_0.53(1)_In_0.47_Ge_2_ were applied to this model. Further details about this structure model are provided in Supporting Information Table S1.

**Figure 5:**
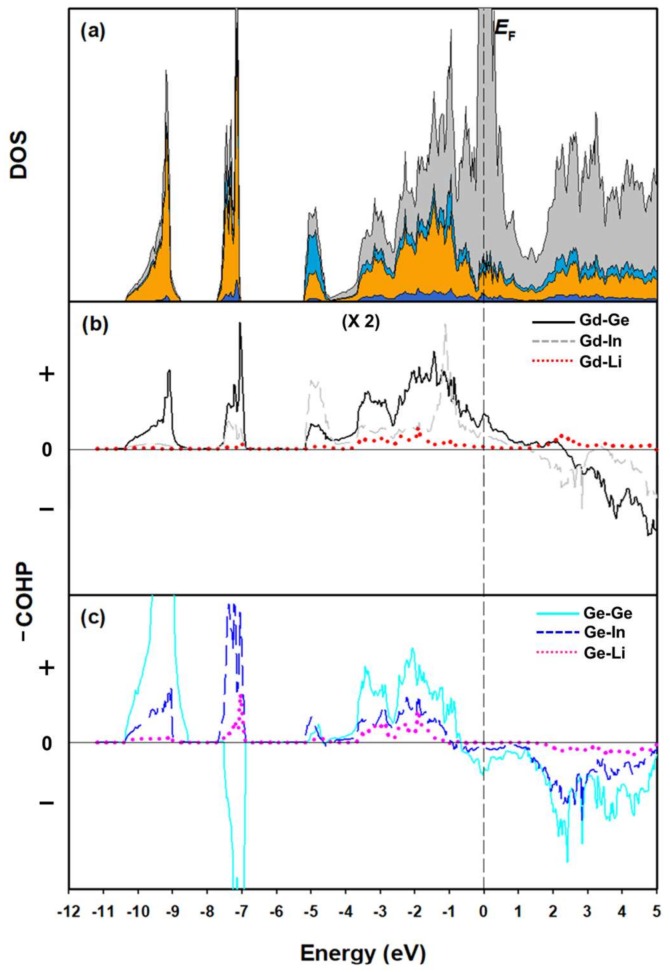
DOS and COHP curves of Gd_2_In_0.5_Li_0.5_Ge_2_. (**a**) TDOS (solid-black line), Gd PDOS (gray region), In PDOS (light-blue region), Ge PDOS (orange region), and Li PDOS (blue region). Six COHP curves represent interatomic interactions (**b**) for the distorted-pentagonal prismatic coordinate environment surrounding the central Gd, and (**c**) among three anionic elements. *E*_F_ (dashed vertical line) is the energy reference (0 eV).

Figure 5a displays the total and partial DOS (TDOS and PDOS) curves of Gd_2_In_0.5_Li_0.5_Ge_2_. The overall features of these DOS curves below *E*_F_ can be divided into three sectors. The lowest region, between ca. −10.5 and −9 eV, shows a large peak that can be mostly attributed to Ge 4*s* states forming the *σ*_s_ bonding interaction within the Ge_2_ dimers, with slight additions of Gd 6*s* and Li 2*s* states also being observed. The region between −7.7 and −7 eV is also mostly contributed by Ge 4*s* states of the Ge_2_ dimers, but forming the *σ*_s_^*^ antibonding interaction. The relatively wider energy region between −5.5 and 0 eV displays a mixed contribution of various orbital states including Gd 4*f*, 5*d*, In 5*p*, Li 2*s*, and Ge 4*p* states. The conduction band region beyond *E*_F_ is dominated by Gd 4*f* and 5*d* states with some contributions from three other anions. A large DOS value at *E*_F_ implies the metallic conductivity of this compound. Overall, no particularly strong orbital mixing of the *s* and *p* states of any individual atom was observed throughout the entire energy window.

The chemical formula of this Li-doped model can be re-written as [(Gd^3+^)_2_(In^3+^)_0.5_(Li^+^)_0.5_][(Ge^3−^)_2_](e^−^)_2_ on the basis of the Zintl-Klemm formalism. Several previously reported isotypic compounds, such as (Gd^3+^)_2_(Mg^2^^+^)_2_(Ge^3−^)_2_(e^−^)_2_ and (Yb^2^^+^)_2_(In^3+^)(Ge^3−^)_2_](e^−^), already followed the same formalism [17]. In our Gd-containing title compound, Gd, In and Li are considered cationic elements donating eight electrons, whereas each Ge forming one covalent bond with the neighboring Ge requires only three more electrons to satisfy the Zintl-Klemm formalism. Therefore, the Ge_2_ dimer should be treated as an isoelectronic to a halogen dimer with a formal charge of 6-. As a result, two extra valence electrons per formula unit remain for the metal-metal bonding in the conduction band. Although the Zintl-Klemm formalism can successfully provide an insight to understand the formal charge of each atom in Gd_2_In_0.5_Li_0.5_Ge_2_, one should keep in mind that this formalism also supplies a rather simplified understanding about the crystal structure since there still exist unneglectable orbital contributions from Gd, In and Li forming the Gd-Ge, In-Ge and Li-Ge interactions in the valence band region as shown in Figure 5b. Figure 5b,c illustrate six COHP curves representing the bonding and antibonding interactions between two neighboring atoms. For clarity, the Gd *f*-orbitals are not included in these COHP curves. First, Figure 5b shows three interactions, including Gd and three anionic elements. Although the magnitude of these COHP curves is doubled for easier comparison with the three other COHP curves shown in Figure 5c, it is clear that rather large contribution from Gd orbitals in the valence band region creates unneglectable interatomic interactions with surrounding anionic elements. In particular, the Gd-Ge COHP curve shows the strongest bonding character among these three COHP curves, which continues up to +1.5 eV beyond *E*_F_. This additional bonding character around *E*_F_ can be used to compensate for the antibonding character of the Ge-Ge COHP curve just below *E*_F_. Second, Figure 5c displays three interatomic interactions among three anionic elements. As one can expect from the selected interatomic distances provided in Table 3, the Ge-Ge COHP curve shows the largest bonding character among three COHP curves in the valence band. In addition, the integrated COHP (ICOHP) values of these three bond, which represent the bonding energy of a particular chemical bond, also prove that the homonuclear Ge-Ge bonding (0.180 eV/bond) is relatively stronger than two other heterogeneous Ge-In (0.098 eV/bond) and Ge-Li (0.027 eV/bond) bonding in the 2D anionic Ge_2_(In/Li) layer, as previously reported (Table 4) [42].

**Figure 6:**
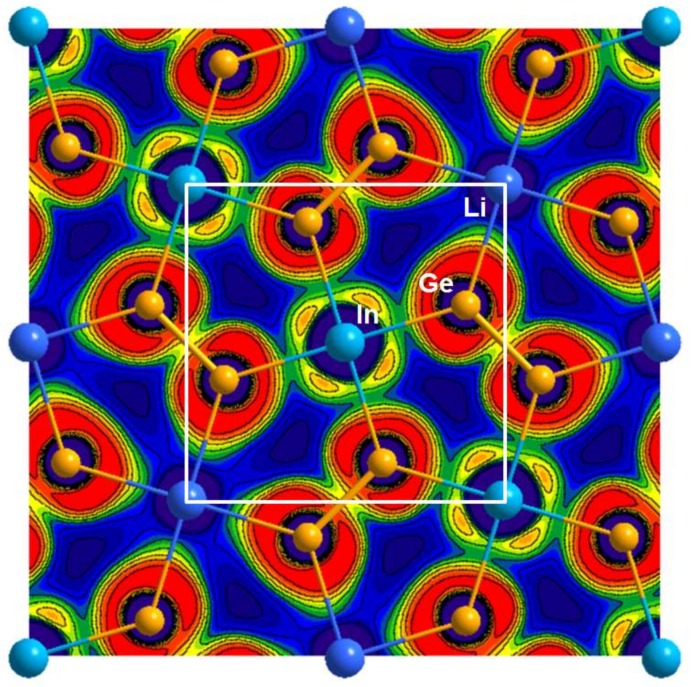
The ELF contour map of Gd_2_In_0.5_Li_0.5_Ge_2_. The (0 0 0) sliced-plane is along the *ab*-plane direction with *z* = 0. The overall diagram is depicted as a filled-and-line contour map, and the 2D Ge_2_(In/Li) layered structure is overlapped on the ELF diagram. The valence electron density color scheme ranges from blue to red (0.0–0.8 e^−^/Å^3^), and values of ELF higher than 0.5 represent the area exceeding free-electron ELF value. Unit cell is outlined in white-color.

Interestingly, this type of the stronger open-shell covalent bond character of the Ge_2_ dimer on the 2D anionic Ge_2_(In/Li) layer can be clearly visualized by an ELF contour map. ELF evaluation is known to visually represent the paired-electron densities observed in bonding pairs as well as lone pairs of electrons [43]. For the current example of Gd_2_In_0.5_Li_0.5_Ge_2_, we particularly chose the (0 0 0) sliced-plane for the ELF mapping analysis since the 2D Ge_2_(In/Li) layer involving the Ge_2_ dimers as well as Li and In atoms were precisely located on the *z* = 0 plane. In Figure 6, the stronger Ge-Ge bonding character, which is previously proven by the COHP curve and the ICOHP value, is represented by the relatively higher ELF value in-between the two Ge atoms. On the other hand, two other relatively weaker bonding characters between Ge-In and Ge-Li bonding are depicted by the lower ELF values.

## 4. Conclusions

We successfully synthesized four quaternary polar intermetallic compounds belonging to the *RE*_2_In_1−*x*_Li*_x_*Ge_2_ (*RE* = La, Nd, Sm, *x* = 0.13(1), 0.28(1), 0.43(1), 0.53(1) by the conventional high-temperature reactions, and their crystal structures were characterized by SXRD analyses. The isotypic crystal structure of the four title compounds adopts the Mo_2_FeB_2_-type structure, and the 2D *RE*_2_In_1-*x*_Li*_x_*Ge_2_ layers can be alternately viewed as the 1:1 intergrowth of two types of hypothetical polyhedral of the *RE*Ge_2_ (AlB_2_-type) and *RE*(In/Li) (CsCl-type) moieties. Interestingly, we revealed that the particular bond distances on the 2D Ge_2_(In/Li) layer gradually decrease as the size of a cation decreases from La^3+^ to Gd^3+^ throughout our title system, and this kind of bond shortening caused by the cationic size reduction should be attributed to the pentagonal-prismatic coordination environment formed by ten anions surrounding the central cation. Further investigations using the late rare-earth metals are in process to see this type of size reduction trend remains. According to the Zintl-Klemm formalism, two extra valence electrons remain on the metal-metal bond in the conduction. In addition, the non-zero DOS value at *E*_F_ implies metallic behavior of the title compounds. Thus, in order to verify the metallic conductivity of the title system, the phase pure product should be obtained first, and the electrical conductivity measurement also need to be performed.

## Figures and Tables

**Table 1:** SXRD data and structure refinement results for the *RE*_2_In_1−_*_x_*Li*_x_*Ge_2_ (*RE* = La, Nd, Sm, Gd; *x* = 0.13(1), 0.28(1), 0.43(1), 0.53(1)) system.

Refined Composition		La_2_In_0.72(1)_Li_0.28_Ge_2_	Nd_2_In_0.87(1)_Li_0.13_Ge_2_	Sm_2_In_0.57(1)_Li_0.43_Ge_2_	Gd_2_In_0.47(1)_Li_0.53_Ge_2_
Formula weight (g/mol)		923.06	917.41	940.93	989.65
Space group; *Z*		*P*4*/mbm*; 2			
Lattice parameters (Å)	*a*	7.6140(3)	7.4368(2)	7.3834(3)	7.2885(3)
	*c*	4.4496(2)	4.3263(10)	4.2704(2)	4.2416(2)
Volume (Å^3^)		257.957	239.121	232.799	225.323
*d*_calcd_ (g/cm^3^)		11.88	12.74	13.42	14.59
Independent reflections		594 (*R*_int_ = 0.0406)	251 (*R*_int_ = 0.0460)	120 (*R*_int_ = 0.0355)	153 (*R*_int_ = 0.0529)
Data/restraints/parameters		594/0/13	251/0/13	120/0/13	153/0/13
*R*^a^ indices (*I* > 2*σ*_I_)	*R*_1_	0.0201	0.0146	0.0161	0.0188
	*wR*_2_	0.0375	0.0309	0.0349	0.0397
GOF on *F*^2^		1.096	1.098	1.249	1.161
Largest differences of peak/hole (e/Å^3^)		1.373/−1.276	2.320/−2.693	0.768/−0.705	2.436/−0.846

^a^ *R*_1_ = Σ||F_o_| − |F_c_||/Σ|F_o_|; *w*R_2_ = (Σ(*w*(F_o_^2^ − F_c_^2^)/Σ(*w*(F_o_^2^)^2^))^1/2^, where *w* = 1/(*σ*^2^*F*_o_^2^ + (A − *P*)^2^ + B – *P*), and *P* = (*F*_o_^2^ + 2*F*_c_^2^)/3; A and B—weight coefficients.

**Table 2:** Atomic coordinates and equivalent isotropic displacement parameters (*U*_eq_ *^a^*) from the SXRD refinements for the *RE*_2_In_1−_*_x_*Li*_x_*Ge_2_ (*RE* = La, Nd, Sm, Gd; *x* = 0.13(1), 0.28(1), 0.43(1), 0.53(1)) system.

Atom	*Wyckoff* Site	Occupation	*x*	*y*	*z*	*U*_eq_ (Å^2^)
La_2_In_0.72(1)_Li_0.28_Ge_2_						
La	4*h*	1	0.1796(1)	0.6796(1)	1/2	0.009(1)
Ge	4*g*	1	0.6179(1)	0.1179(1)	0	0.011(1)
In/Li	2*a*	0.72(1)/0.28	0	0	0	0.018(1)
Nd_2_In_0.87(1)_Li_0.13_Ge_2_						
Nd	4*h*	1	0.1791(1)	0.6791(1)	1/2	0.007(1)
Ge	4*g*	1	0.6197(1)	0.1197(1)	0	0.008(1)
In/Li	2*a*	0.87(1)/0.13	0	0	0	0.014(1)
Sm_2_In_0.57(1)_Li_0.43_Ge_2_						
Sm	4*h*	1	0.1785(1)	0.6785(1)	1/2	0.008(1)
Ge	4*g*	1	0.6209(1)	0.1209(1)	0	0.009(1)
In/Li	2*a*	0.57(1)/0.43	0	0	0	0.015(1)
Gd_2_In_0.47(1)_Li_0.53_Ge_2_						
Gd	4*h*	1	0.1792(1)	0.6792(1)	1/2	0.008(1)
Ge	4*g*	1	0.6213(1)	0.1213(1)	0	0.010(1)
In/Li	2*a*	0.47(1)/0.53	0	0	0	0.010(1)

*^a^* *U*_eq_ is defined as one-third of the trace of the orthogonalized *U*_ij_ tensor.

**Table 3:** Selected interatomic distances (Å) for the *RE*_2_In_1−_*_x_*Li*_x_*Ge_2_ (*RE* = La, Nd, Sm, Gd; *x* = 0.13(1), 0.28(1), 0.43(1), 0.53(1)) system.

Atomic Pair		Bond Distance (Å)		
	La_2_In_0.72(1)_Li_0.28_Ge_2_	Nd_2_In_0.87(1)_Li_0.13_Ge_2_	Sm_2_In_0.57(1)_Li_0.43_Ge_2_	Gd_2_In_0.47(1)_Li_0.53_Ge_2_
*RE*-*RE* (shorter)	3.8670(2)	3.7681(3)	3.7273(6)	3.6942(5)
*RE*-*RE* (longer)	3.9552(2)	3.8649(3)	3.8398(6)	3.7876(5)
*RE*-Ge (shorter)	3.1157(3)	3.0246(4)	2.9911(7)	2.9543(7)
*RE*-Ge (longer)	3.2092(2)	3.1319(4)	3.1026(8)	3.0776(7)
*RE*-In/Li	3.5737(1)	3.4845(2)	3.4541(4)	3.4362(3)
Ge-Ge	2.5384(8)	2.5185(7)	2.5252(14)	2.4998(12)
Ge-In/Li	3.0448(3)	2.9649(5)	2.9378(10)	2.8985(9)

**Table 4:** ICOHP values (ev/bond) of the six selected interatomic interactions in the *RE*_2_In_1−_*_x_*Li*_x_*Ge_2_ (*RE* = La, Nd, Sm, Gd; *x* = 0.13(1), 0.28(1), 0.43(1), 0.53(1)) system.

Atomic Pair	Gd-Ge	Gd-In	Gd-Li	Ge-Ge	Ge-In	Ge-Li
ICOHP	0.088	0.06	0.009	0.180	0.098	0.027

## Supplementary Materials

The following are available online at http://www.mdpi.com/1996-1944/11/4/495/s1, Table S1: Detailed information of a hypothetical structural model Gd_2_In_0.5_Li_0.5_Ge_2_.

## References

[1] Mozharivskyj Y., Pecharsky A.O., Pecharsky V.K., Miller G.J. (2005). On the High-Temperature Phase Transition of Gd_5_Si_2_Ge_2_. J. Am. Chem. Soc..

[2] Wang H., Wang F., Jones K., Miller G.J. (2011). Chemical Pressure and Rare-Earth Orbital Contributions in Mixed Rare-Earth Silicides La_5–*x*_Y*_x_*Si_4_ (0 ≤ *x* ≤ 5). Inorg. Chem..

[3] Wu L.-M., Kim S.-H., Seo D.-K. (2005). Electron-Precise/Deficient La_5−*x*_Ca*_x_*Ge_4_ (3.4 ≤ *x* ≤ 3.8) and Ce_5−*x*_Ca*_x_*Ge_4_ (3.0 ≤ *x* ≤ 3.3):  Probing Low-Valence Electron Concentrations in Metal-Rich Gd_5_Si_4_-type Germanides. J. Am. Chem. Soc..

[4] Morellon L., Blasco J., Algarabel P.A., Ibarra M.R. (2000). Nature of the first-order antiferromagnetic-ferromagnetic transition in the Ge-rich magnetocaloric compounds Gd_5_(Si*_x_*Ge_1−*x*_)_4_. Phys. Rev. B.

[5] Levin E.M., Pecharsky V.K., Gschneidner K.A. (2001). Spontaneous generation of voltage in Gd_5_(Si*_x_*Ge_4*−x*_) during a first-order phase transition induced by temperature or magnetic field. Phys. Rev. B.

[6] Morellon L., Algarabel P.A., Ibara M.R., Blasco J., Garcia-Landa B. (1998). Magnetic-field-induced structural phase transition in Gd_5_(Si_1.8_Ge_2.2_). Phys. Rev. B.

[7] Morellon L., Stankiewicz J., Garcia-Landa B., Algarabel P.A., Ibarra M.R. (1998). Giant magnetoresistance near the magnetostructural transition in Gd_5_(Si_1.8_Ge_2.2_). Appl. Phys. Lett..

[8] Levin E.M., Pecharsky V.K., Gschneidner K.A. (1999). Magnetic-field and temperature dependencies of the electrical resistance near the magnetic and crystallographic first-order phase transition of Gd_5_(Si_2_Ge_2_). Phys. Rev. B.

[9] Levin E.M., Pecharsky V.K., Gschneidner K.A., Tomlinson P. (2000). Magnetic field and temperature-induced first-order transition in Gd_5_(Si_1.5_Ge_2.5_): A study of the electrical resistance behavior. J. Magn. Magn. Mater..

[10] Percharsky V.K., Gschneidner K.A. (1997). Giant Magnetocaloric Effect in Gd_5_(Si_2_Ge_2_). Phys. Rev. Lett..

[11] Pecharsky V.K., Gschneidner K.A. (1997). Tunable magnetic regenerator alloys with a giant magnetocaloric effect for magnetic refrigeration from ~20 to ~290 K. Appl. Phys. Lett..

[12] Wang H., Misra S., Wang F., Miller G.J. (2010). Structural and Magnetic Characteristics of Gd_5_Ga*_x_*Si_4−*x*_. Inorg. Chem..

[13] Misra S., Poweleit E.T., Miller G.J. (2009). On the Crystal Structure, Metal Atom Site Preferences and Magnetic Properties of Nd_5–*x*_Er*_x_Tt*_4_ (*Tt* = Si or Ge). Z. Anorg. Allg. Chem..

[14] Miller G.J. (2006). Complex rare-earth tetrelides, *RE*_5_(Si*_x_*Ge_1−*x*_)_4_: New materials for magnetic refrigeration and a superb playground for solid state chemistry. Chem. Soc. Rev..

[15] Nam G., Jeon J., Kim Y., Kang S., Ahn K., You T.-S. (2013). Combined effect of chemical pressure and valence electron concentration through the electron-deficient Li substitution on the *RE*_4_LiGe_4_ (*RE* = La, Ce, Pr, and Sm) system. J. Solid State Chem..

[16] Rieger W., Nowotny H., Benesovsky F. (1964). Die Kristallstruktur von Mo_2_FeB_2_. Monatsh. Chem..

[17] Choe W., Miller G.J., Levin E.M. (2001). Crystal structure and magnetism of Gd_2_MgGe_2_. J. Alloy. Compd..

[18] Zaremba V.I., Kaczorowski D., Nychyporuk G.P., Rodewald U.C., Pöttgen R. (2004). Structure and physical properties of *RE*_2_Ge_2_In (*RE* = La, Ce, Pr, Nd). Solid State Sci..

[19] Tobash P.H., Lins D., Bobev S., Lima A., Hundley M.F., Thompson J.D., Sarrao J.L. (2005). Crystal Growth, Structural, and Property Studies on a Family of Ternary Rare-Earth Phases *RE*_2_InGe_2_ (*RE* = Sm, Gd, Tb, Dy, Ho, Yb). Chem. Mater..

[20] Zaremba V.I., Tyvanchuk Y.B., Stepien-Damm J. (1997). Crystal structure of diytterbium digermanium indide, Yb_2_Ge_2_In. Z. Kristallogr. New Cryst. Struct..

[21] Zaremba V.I., Stepien-Damm J., Nichiporuk G.P., Tyvanchuk Y.B., Kalychak Y.M. (1998). Structure of R_2_Ge_2_In crystals, where R is a rare-earth metal. Crystallogr. Rep..

[22] Steinberg G., Schuster H.-U. (1979). Ternary Silieides of Lithium with Yttrium or Neodymium in a Modified U_3_Si_2_-Type Structure. Z. Naturforsch..

[23] Dhar S.K., Manfrinetti P., Palenzona A. (1997). Magnetic ordering in CeMg_2_Si_2_ and Ce_2_MgSi_2_. J. Alloys Compd..

[24] Kranenberg C., Mewis A. (2000). Darstellung und Kristallstrukturen von *Ln*_2_Al_3_Si_2_ and *Ln*_2_AlSi_2_ (*Ln*: Y, Tb–Lu). Z. Anorg. Allg. Chem..

[25] Dhar S.K., Manfrinetti P., Palenzona A., Kimura Y., Kozaki M., Onuki Y., Takeuchi T. (1999). Magnetic, transport and thermal behaviour of *R*_3_Si_2_, *R*_2_YSi_2_ (*R* = La and Ce), Ce_2_ScSi_2_ and Ce_2_Sc_3_Si_4_. Physics B.

[26] Kraft R., Pöttgen R. (2005). Syntheses and Crystal Structure of the Ternary Silicides *RE*_2_Si_2_Mg (*RE* = Y, La–Nd, Sm, Gd–Lu) and Structure Refinement of Dy_5_Si_3_. Monatsh. Chem..

[27] Kraft R., Pöttgen R. (2004). Ternary Germanides *RE*_2_Ge_2_Mg (*RE* = Y, La–Nd, Sm, Gd, Tb). Monatsh. Chem..

[28] Dinges T., Rodewald U.C., Matar S.F., Eckert H., Pöttgen R. (2009). New Ternary Silicide LiRh_2_Si_2_—Structure and Bonding Peculiarities. Z. Anorg. Allog. Chem..

[29] Bruker (2007). APEX2.

[30] Bruker (2002). SAINT.

[31] Sheldrick G.M. (2003). SADABS.

[32] Gelato L.M., Parthé E. (1987). STRUCTURE TIDY—A computer program to standardize crystal structure data. J. Appl. Crystallogr..

[33] Andersen O.K. (1975). Linear methods in band theory. Phys. Rev. B..

[34] Andersen O.K., Jepsen O. (1984). Explicit, first-principles tight-binding theory. Phys. Rev. Lett..

[35] Andersen O.K. (1986). Minimal basis sets in the linear muffin-tin orbital method: Application to the diamond-structure crystals C, Si, and Ge. Phys. Rev. B..

[36] Jepsen O., Burkhardt A., Andersen O.K. (1999). The TB-LMTO-ASA Program, Version 4.7.

[37] Andersen O.K., Jepsen O., Glötzel D., Bassani F., Fumi F., Tosi M. (1985). Highlights of Condensed Matter Theory.

[38] Jepsen O., Andersen O.K. (1995). Calculated electronic structure of the sandwich *d*^1^ metals LaI2 and CeI2: Application of new LMTO techniques. Z. Phys. B Condens. Matter.

[39] Blöchl P.E., Jepsen O., Andersen O.K. (1994). Improved tetrahedron method for Brillouin-zone integrations. Phys. Rev. B.

[40] Zachariasen W.H. (1949). Crystal chemical studies of the 5*f*-series of elements. VIII. Crystal structure studies of uranium silicides and of CeSi_2_, NpSi_2_, and PuSi_2_. Acta Crysallogr..

[41] Shannon R.D. (1976). Revised effective ionic radii and systematic studies of interatomic distances in halides and chalcogenides. Acta Crystallogr..

[42] Dronskowski R., Blöchl P.E. (1993). Crystal orbital Hamilton populations (COHP): Energy-resolved visualization of chemical bonding in solids based on density-functional calculations. J. Phys. Chem..

[43] Becke A.D., Edgecombe K.E. (1994). Classification of chemical bonds based on topological analysis of electron localization functions. Nature.

