# Supplementary material for: Structure and Chemical Bonding of the Li-Doped Polar Intermetallic RE2In1−xLixGe2 (RE = La, Nd, Sm, Gd; x = 0.13, 0.28, 0.43, 0.53) System
Source: Materials (Basel). 2018 Mar 26;11(4):495. doi: 10.3390/ma11040495 (PMC5951341; doi:10.3390/ma11040495)
Supplement: Supplementary file 1 [file materials-11-00495-s001.docx]

Supporting Information

Structure and Chemical Bonding of the Li-Doped Polar Intermetallic *RE*_2_In_1-_*_x_*Li*_x_*Ge_2_ (*RE* = La, Nd, Sm, Gd; x = 0.13, 0.28, 0.43, 0.53) System

Junsu Lee, Jieun Jeon and Tae-Soo You^*^

Department of Chemistry, Chungbuk National University, Cheongju, Chungbuk 28644, Republic of Korea

**Table S1.** Detailed information of a hypothetical structural model Gd_2_In_0.5_Li_0.5_Ge_2_.

| **Chemical formula** | | | **Gd_2_In_0.5_Li_0.5_Ge_2_** | | |  |
| --- | --- | --- | --- | --- | --- | --- |
| Space group | | | *Pmma* (No.51) | | |  |
| Unit cell dimensions (Å) | | | *a* = 10.3075  *b* = 8.4832  *c* = 10.3075 | | |  |
| Volume (Å^3^) | | | 901.293 | | |  |
| Atomic coordinates | | | | | |  |
| Atom | *Wyckoff* site | *x* | | *y* | *z* | |
| Gd1 | 8*l* | 0.0708 | | 1/4 | 1/4 | |
| Gd2 | 4*k* | 1/4 | | 1/4 | 0.5708 | |
| Gd3 | 4*k* | 1/4 | | 1/4 | 0.9292 | |
| Ge1 | 4*j* | 0.6287 | | 1/2 | 1/4 | |
| Ge2 | 4*i* | 0.6287 | | 0 | 1/4 | |
| Ge3 | 2*f* | 1/4 | | 1/2 | 0.1287 | |
| Ge4 | 2*f* | 1/4 | | 1/2 | 0.3713 | |
| Ge5 | 2*e* | 1/4 | | 0 | 0.1287 | |
| Ge6 | 2*e* | 1/4 | | 0 | 0.3713 | |
| In1 | 2*c* | 0 | | 0 | 1/2 | |
| In2 | 2*b* | 0 | | 1/2 | 0 | |
| Li1 | 2*d* | 0 | | 1/2 | 1/2 | |
| Li2 | 2*a* | 0 | | 0 | 0 | |
